# Staphyloma 3D images created by a low-cost portable device

**DOI:** 10.5935/0004-2749.202100110

**Published:** 2021

**Authors:** Marcelo Bezerra Diógenes, Nayara Queiroz Cardoso Pinto, Karlos Ítalo Souza Viana, Dácio Carvalho Costa

**Affiliations:** 1 Ophthalmology Department, Hospital Geral de Fortaleza, Fortaleza, CE, Brazil; 2 Ophthalmology Department, Hospital Universitário Walter Cantídio, Fortaleza, CE, Brazil

Dear Editor,

Portable devices are increasingly being used to acquire retinal images as a result of technological advancements and the increased availability of imaging equipment, such as smartphones^(1,2)^. Retinal pictures acquired by smartphones help identify various diseases, such as glaucoma, age-related macular degeneration, diabetic retinopathy, and retinopathy of prematurity^(2)^.

Teleophthalmology, with the use of these devices, can help screen and refer patients with ocular diseases to a trained specialist.^(3,4)^ It can also help create efficient long-distance follow-up systems, promoting health care accessibility to underserved populations.^(2,3)^

Despite the recent progress in imaging equipment, however, poor image quality still a limitation.^(1)^ This obstacle may result from insufficient training in image capture and from inadequate technology^(3)^. Therefore, we developed a photographic model that is easy to use and capable of acquiring three-dimensional (3D) retinal imaging.

**Figure 1:**
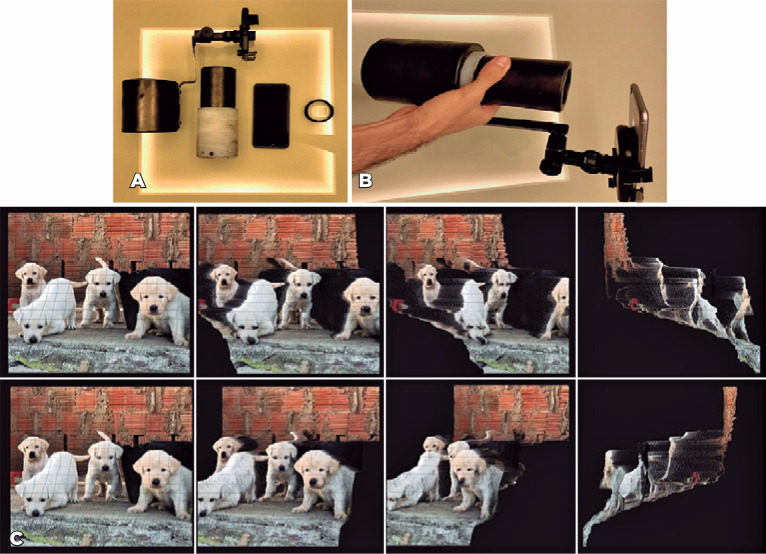
(A) Two cylindrical nylon molds, a regular car phone holder, the smartphone with two 12-megapixel cameras (iPhone 7 Plus™; Apple Inc., Cupertino, CA, USA), and a 20-diopter lens (Volk Optical, Inc., Mentor, OH, USA). (B) The iPhone 7 Plus™ aligned with the 20-diopter lens through the prototype device. (C) Use of the Focos application™ with various perks to exhibit the effectiveness of the software.

We built a low-cost prototype capable of aligning a smartphone with two 12-megapixel cameras (iPhone 7 Plus™; Apple Inc., Cupertino, CA, USA) and a 20-diopter lens (Volk Optical, Inc., Mentor, OH, USA) through two cylindrical nylon molds and a regular car phone holder^(2)^ (Figure 1).

**Figure 2:**
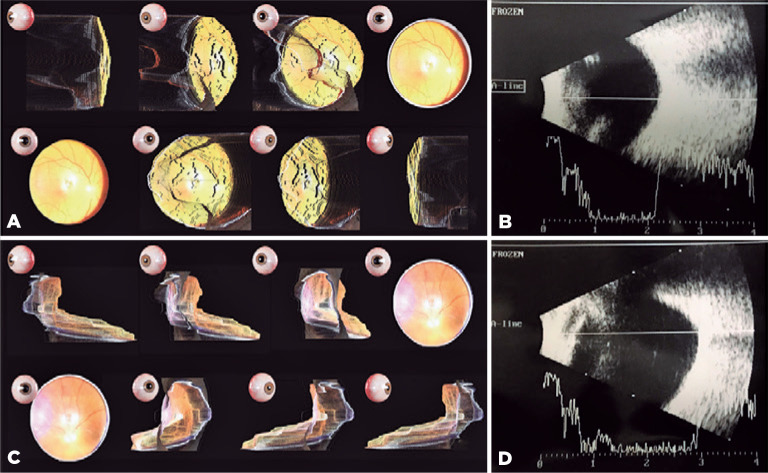
(A) Three-dimensional pictures of the emmetrope eye. (B) Echography of the emmetrope eye. (C) Three-dimensional pictures of the myopic eye. Note the position of the optic nerve within the staphyloma. (D) Echography of the myopic eye. Note the shadow of the optic nerve along the staphyloma topography. The “effect” perk of the Focos application™ allows the user to rotate the image (A,C) 180 degrees, which gives the appearance of stereopsis.

After obtaining ethical approval for the study, we tested the portable device on a myopic eye (11 diopters) and on an emmetropic eye. To capture the pictures, the eyes were dilated with 1% tropicamide, and Focos™ by Xiaodong Wang (created in 2017) was configured with the following perks: continuous shooting mode, export size of 1536 × 2048 and flashlight set to torch. After image capture, we used the Focos™ application’s “effect” perk to process the images (Figure 2).

After pictures were acquired and processed, the patients were examined by five ophthalmologists, one retina specialist, and four general ophthalmologists. Each clinician performed ocular echography on each studied eye. The ophthalmologists then compared the results of echography with the processed fundus images.

We were able to acquire 3D fundus images by using our smartphone model. In analyzing the pictures, the four general ophthalmologists and the retina specialist were able to identify a staphyloma on the myopic eye, but only the retina specialist was able to locate the staphyloma with ocular echography. The echography performed by the five ophthalmologists’ revealed similar axial lengths in each eye: 29.5 mm in the myopic eye and 22.3 mm in the emmetropic eye (Figure 2).

In comparison with commercially available fundus devices, the quality of images obtained with a smartphone is the worst^(1)^. Glare and improper exposure are some of the major reasons for poor quality. To decrease glare and patient discomfort, we painted the molds matte black and placed a Micropore strip over the light source. Image quality is improved when the operator is trained in the use of the camera and has a steady hand^(3)^. To ease training, the nylon molds were fitted with a screw-thread device that enabled focus with a slow and firm shift of the distance between the eye and the 20-diopter lens.

Delay in proper treatment of ocular cancer is a national health problem in Brazil.^(4)^ Devices that can acquire images with stereopsis might have more significant screening potential for identifying intraocular tumors, retinal detachments, and staphylomas that would go unnoticed by clinicians without proper training in stereopsis testing or ocular echography. Unfortunately, these devices are not used for screening because they are not portable and are costly. Because a camera system with two positions corresponding to the two eye positions can acquire images with stereopsis^(5)^, we used a smartphone with two cameras to capture 3D retinal pictures.

Despite some glare and artifacts common on pictures of the fundus taken by smartphones^(1)^, our 3D images have good reliability. In analyzing the optic nerve in the image of the myopic eye, we were able to verify that it was located superiorly within the staphyloma, as which corresponded to the findings on echography (Figure 2).

Ocular oncologists and retina specialists are trained to perform specific examinations, such as echography, and to handle complex vision and life-threatening illnesses, and the numbers of these clinicians are too low to meet the country’s needs. We believe that the staphyloma was not identified by the four general ophthalmologists because of their inexperience in ocular echography. Therefore, an easy to use technology capable of screening for ocular diseases is necessary to improve health care through telemedicine^(3)^.

The use of a smartphone to obtain pictures of the fundus with stereopsis has not been previously reported and must be validated for screening purposes. We believe that our device may be useful in communities without access to ocular echography or trained specialists.
